# Glenoid morphology in patients undergoing reverse total shoulder arthroplasty due to fracture

**DOI:** 10.1007/s00402-025-05977-8

**Published:** 2025-07-15

**Authors:** Jackson S. Hamersly, Mason D. Tippy, James E. Slaven, Yohan Jang, Lauren M. Ladd, Mark T. Dillon

**Affiliations:** 1 Indiana University School of Medicine, Indianapolis, Indiana, United States; 2 Department of Radiology, Indiana University School of Medicine, Indianapolis, Indiana, United States; 3 Department of Biostatistics and Health Data Science, Indiana University School of Medicine, Indianapolis, Indiana, United States; 4 Department of Orthopaedic Surgery, Indiana University School of Medicine, Indianapolis, Indiana, United States

**Keywords:** Reverse total shoulder arthroplasty, Computed tomography, Glenoid anatomy, RSA angle, Trauma

## Abstract

**Introduction:**

Glenoid morphology in patients undergoing reverse total shoulder arthroplasty (rTSA) due to arthritis has been previously studied; however, it has not been as thoroughly evaluated in fracture populations. The purpose of this study is to utilize pre-operative computed tomography (CT) scans to better understand the glenoid anatomy of those patients undergoing rTSA due to fracture.

**Materials and methods:**

Patients over the age of 18 who underwent rTSA for proximal humerus fractures from January 1, 2015 to October 31, 2023 at two university health system affiliated hospitals were included if they had a CT scan available for review and image reconstruction. Patients were excluded if a pathologic fracture was identified, surgery was performed greater than 6 weeks after the initial injury, surgery was a conversion or revision surgery, or if a glenoid fracture was present. Glenoid version and reverse shoulder arthroplasty (RSA) angles were measured by a musculoskeletal fellowship-trained radiologist and a shoulder and elbow fellowship-trained orthopaedic surgeon and averaged for final values. Glenoid morphologies were determined using the Walch and Favard classifications.

**Results:**

A total of 53 patients with a mean age of 70.4 years (range 36.6–91.2) were included in this study, 84.9% of which were female. Walch A1 glenoid morphology was noted in 92.5% of patients, and Favard E0 morphology was present in 98.1% of patients. Median glenoid version was 3° of retroversion. Median RSA angle was 19°. Of note, 37.7% of patients had a RSA angle of ≥ 20°.

**Conclusions:**

Patients undergoing rTSA for fracture may not have significant glenoid deformity from arthritic wear. However, surgeons should be aware of variations in glenoid version and RSA angle. In this study population, over one-third of patients had a RSA angle of ≥ 20°. Thus, surgeons should take these findings into account when performing rTSA for fracture.

## Introduction

Proximal humerus fractures are a common injury in elderly populations. It has been reported that proximal humerus fractures are the third most common fracture in patients over 65 years of age, behind only hip and distal radius fractures [1]. The majority of these injuries can be treated non-operatively [2]. When operative treatment is deemed necessary, shoulder hemiarthroplasty has historically been the preferred treatment if the fracture pattern is not amenable to open reduction and internal fixation. Within the last decade, however, reverse total shoulder arthroplasty (rTSA) has gained popularity as the treatment of choice in elderly patients with proximal humeral fractures [3]. RTSA has been shown to produce reliable and improved results compared to hemiarthroplasty for fracture indications [4–8].

When performing rTSA, surgeons must carefully evaluate the patient’s glenoid morphology, including deformity due to arthritis and the angle the glenoid is positioned relative to the scapular body, so that the baseplate and glenosphere may be precisely placed in order to avoid poor outcomes [9–14]. Glenoid version is the angle of the glenoid with respect to the scapular body measured in the axial plane. Historically, glenoid inclination was utilized to assess the angulation of the glenoid in the coronal plane. In 2019, Boileau et al. described the reverse shoulder arthroplasty (RSA) angle for a more clinically relevant assessment of glenoid inclination [15], which better identifies the correction needed to appropriately align the glenoid component with respect to the inferior glenoid and avoid superior tilt. Glenoid morphology is most often characterized by the Walch classification, which evaluates osseous wear in the axial plane due to osteoarthritis [16], and by the Favard classification that evaluates bone loss in the coronal plane, most often due to rotator cuff arthropathy [17].

While the benefit of pre-operative computed tomography (CT) scans is well established in the elective setting when performing rTSA [18–21], they may be less frequently obtained in a trauma setting as indications for advanced imaging are not agreed upon [22]. Furthermore, when a CT is obtained in patients with trauma, it is often done so with the intent of evaluating the fracture pattern of the proximal humerus rather than studying glenoid anatomy. As a result, surgeons may be less likely to utilize existing software for rTSA preoperative planning in the trauma setting, which may allow for the more accurate placement of the glenoid component in such cases. To this end, the purpose of this study is to utilize pre-operative CT scans of patients undergoing rTSA for fracture indications to evaluate glenoid anatomy through assessment of morphology, as well as version and RSA angle.

## Methods

### Patients

**Fig. 1:**
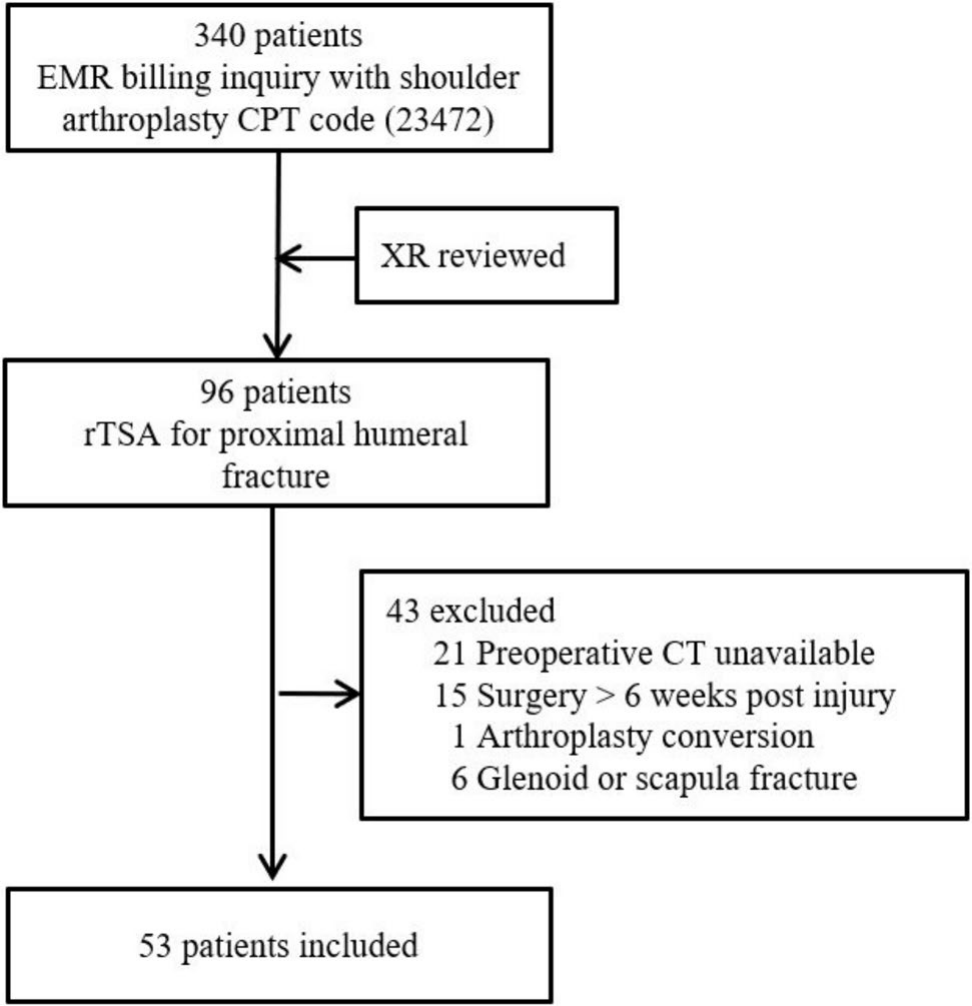
Flowchart of selection of patients for inclusion in present study. *EMR* electronic medical record, *CPT* current procedural terminology, *rTSA* reverse total shoulder arthroplasty

After obtaining institutional review board approval and in compliance with Health Insurance Portability and Accountability Act regulations, we performed a retrospective review of patients over the age of 18 years known to have undergone rTSA between the dates of January 1, 2015 and October 31, 2023 at one of two university-affiliated hospitals. An initial cohort of 340 patients were identified from an electronic medical record billing inquiry using common procedural terminology code for shoulder arthroplasty (23,472). Radiographs were screened to identify patients who underwent rTSA for treatment of a proximal humeral fracture (n = 96), and the electronic medical record system was reviewed to apply exclusion criteria: preoperative CT imaging unavailable for review (n = 21), presence of a pathologic fracture, surgery performed greater than 6 weeks after initial injury (n = 15), surgery performed for arthroplasty conversion or revision (n = 1), or presence of a glenoid or scapula fracture (n = 6) **(**Fig. 1**)**.

### Imaging analysis

Routine CT scans of the shoulder or chest were reviewed and reconstructed into standardized axial and coronal planes with respect to the glenohumeral joint. Pre-existing osteoarthritis of the glenohumeral joint was assessed by a musculoskeletal fellowship-trained radiologist according to the Samilson and Prieto classification [23, 24]. Glenoid version and glenoid inclination were independently measured by a musculoskeletal fellowship-trained radiologist and a shoulder fellowship-trained orthopaedic surgeon.

**Fig. 2:**
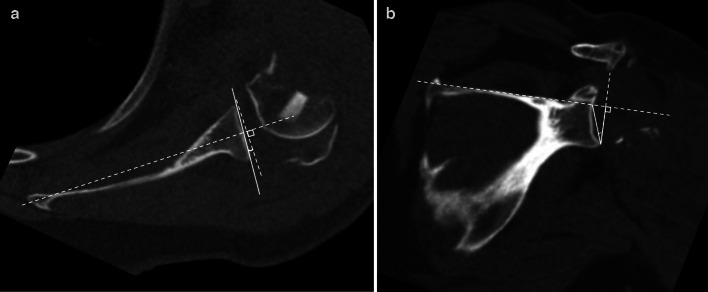
**a** Axial CT image demonstrating Glenoid Version measurement by the Friedman line technique. **b** Coronal CT image showing the reverse shoulder arthroplasty (RSA) angle of glenoid inclination by Boileau technique

Glenoid version measurements were obtained using the Friedman line technique [25]. This includes selecting an axial image at the mid aspect of the glenoid, drawing the Friedman line (a line from the medial margin of the scapula that bisect the glenoid), the line of neutral glenoid version (a line perpendicular to the Friedman line at the level of the glenoid), and the glenoid line (a line connecting the anterior and posterior glenoid rim. The glenoid version is then measured as the acute angle between the glenoid line and the line of neutral glenoid version (Fig. 2a).

Glenoid inclination measurements were obtained using the reverse shoulder arthroplasty (RSA) angle [15]. The RSA angle is similar to glenoid version in that 3 lines are required to create the angle. First, a line along the superior margin of the scapula in plane with the supraspinatus fossa floor is drawn, followed by a line perpendicular to this with its inferior margin contacting the inferior glenoid rim, and a final line connecting the inferior glenoid rim and the point at which the first (transverse) line transects the glenoid fossa. The RSA angle is then measured as the angle between the latter two of the above-described lines (Fig. 2b**)**.

Glenoid morphology was also assessed using the Walch and Favard classifications. The Walch classification assesses the configuration and osseous wear of the glenoid in the axial plane, which is important in the setting of osteoarthritic wear [16, 26]. The Favard classification describes common patterns of glenoid erosion in the coronal plane, initially described in the setting of rotator cuff arthropathy, ranging from normal (E0) to central (E1), eccentric superior (E2 and E3), and inferior (E4) osseous wear [27, 28]. Disagreements in glenoid morphology were adjudicated with consensus review between both interpreting physicians.

### Statistical analysis

Equivalence tests were performed to determine if the two raters had equivalent ratings for the glenoid version and RSA angles, using clinically significant bounds of 2.5° (-2.5,2.5) and therefore establishing a tolerance of 5° for each measurement [29]. All analytic assumptions were verified. Analyses were performed using SAS v9.4 (SAS Institute, Cary, NC) with P < 0.05 indicating statistical significance. Independently obtained data points from manual measurements were aggregated after statistical analysis and measurement data are reported as mean and median between 2 raters after equivalence tests confirmed equivalent ratings.

## Results

**Fig. 3:**
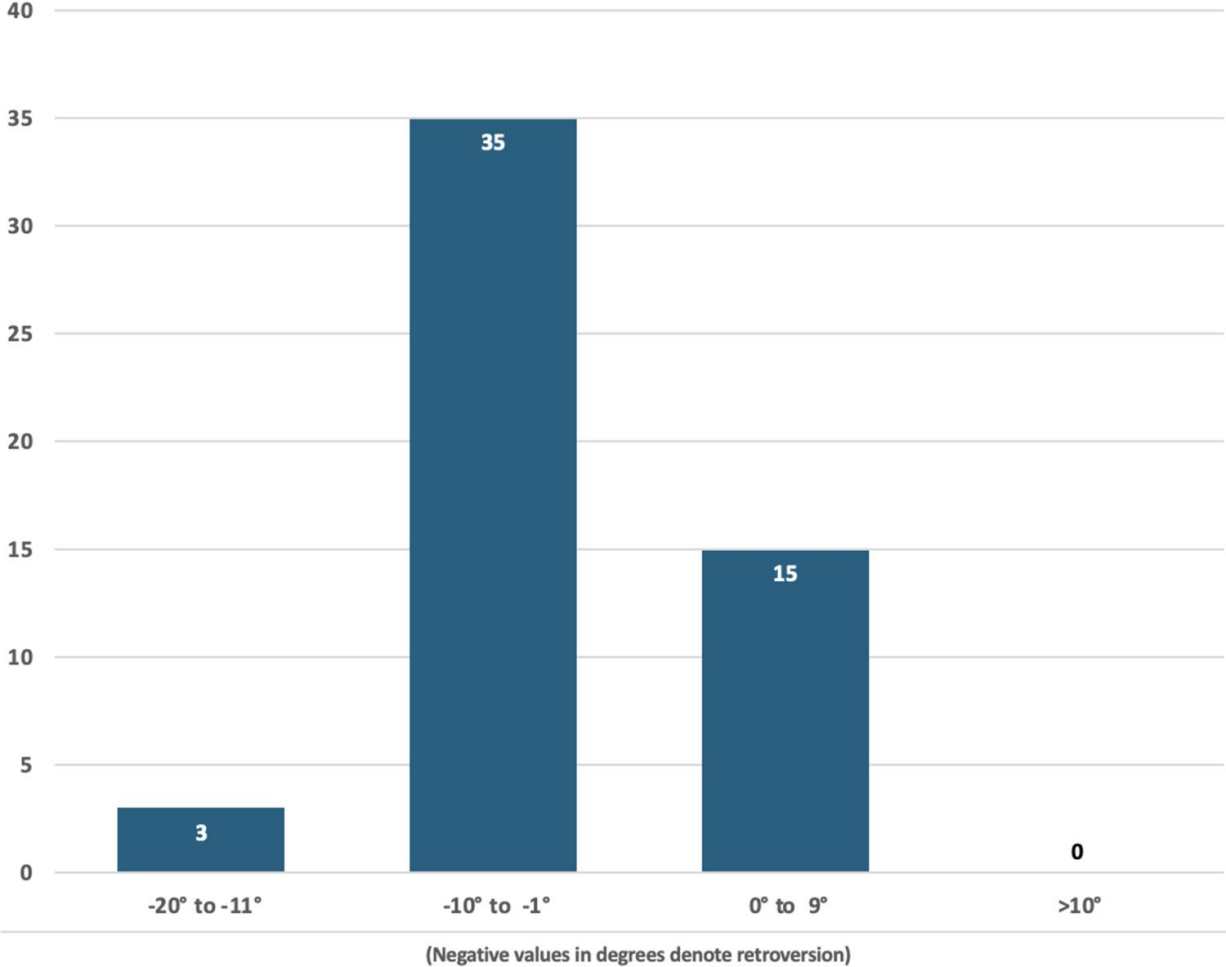
Graph demonstrating the number of patients in each group of Glenoid Version, grouped by severity in both anteversion (positive angle) and retroversion (negative angle)

**Fig. 4:**
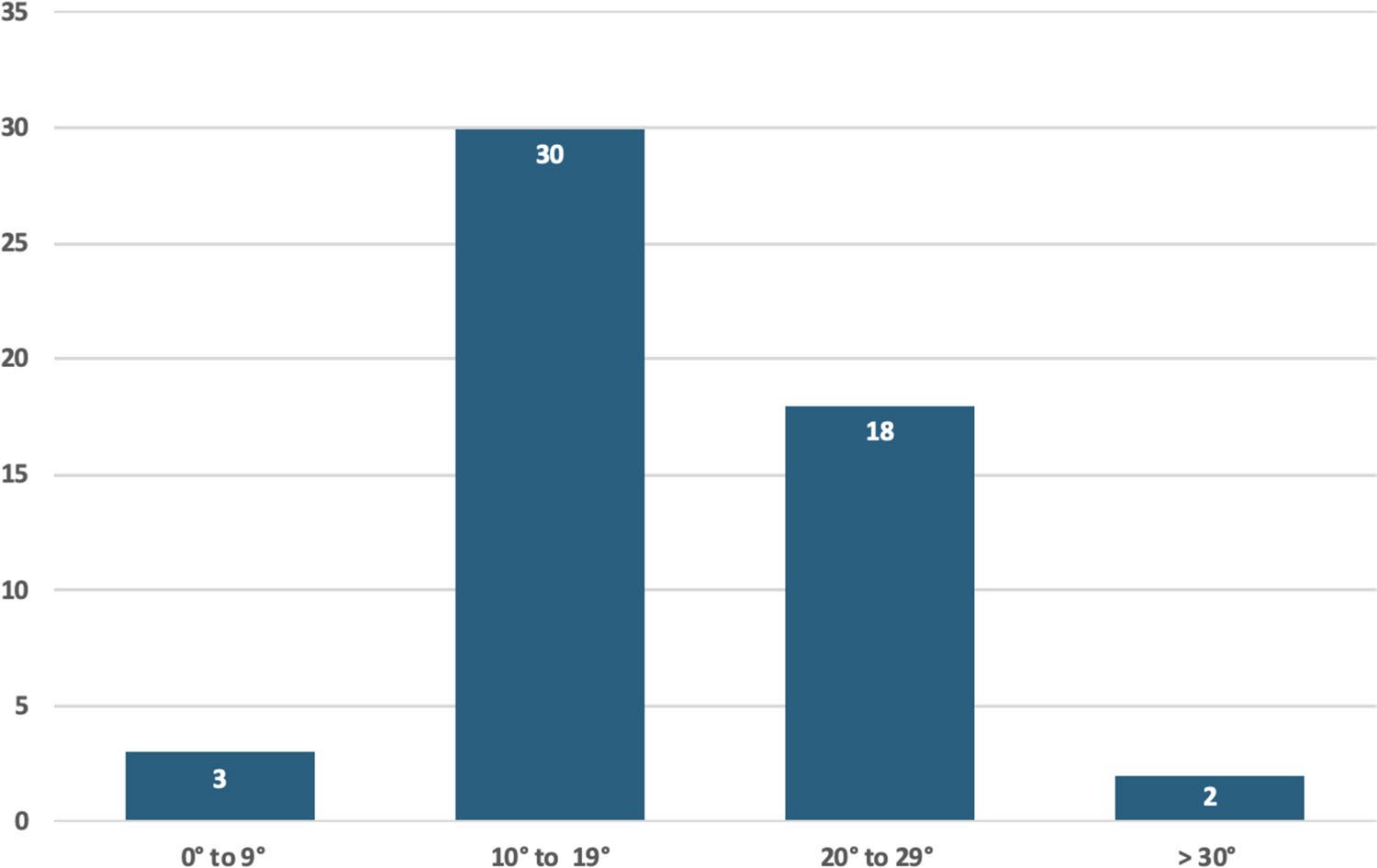
Graph demonstrating the number of patients in each group of RSA angles, grouped by severity

In total, 53 patients were included in the study with a mean age of 70.4 years (range 36.6–91.2), of which 85% were female and 15% were male. Pre-existing arthritis was present in 74% of patients (58% mild, 9% moderate, 6% severe). It was noted that 49 patients (92.5%) had Walch A1 glenoid. There was one Walch A2, B1, B2, and B3 glenoid (1.9% each). Fifty-two patients (98.1%) had a Favard E0 glenoid and one patient had an E1 glenoid. Median glenoid version of the averaged measurements between the two raters was 3° of retroversion, with only three patients (5.7%) having more than 10° of retroversion (Fig. 3). Median RSA angle of averaged measurements between two raters was 19°. Thirty patients (57%) had a RSA angle of 10–19°, and 20 patients (37.7%) had a RSA angle of 20° or greater **(**Fig. 4**)**. The mean difference in glenoid version and RSA angle were 0.66° (95% confidence interval (CI) 1.58,0.26) and 0.45° (95% CI 2.22,1.32), respectively. The equivalence test demonstrated equivalent ratings between the two raters (glenoid version, p = 0.0015 and RSA angle, p = 0.0287) (Table 1).

**Table 1:** Equivalence testing for measurements of Glenoid Version and RSA Angle

	Mean difference in degrees (95% CI)	Equivalence test p-value (2.5 degrees bounds)
Glenoid Version	0.66 (-1.58, 0.26)	0.**0007**
Glenoid Inclination (RSA angle)	0.45 (-2.22, 1.32)	0.**0287**

Values are mean differences (95% confidence intervals) between the raters, with p-values from overall equivalence tests

## Discussion

This study demonstrates a low rate of pre-existing deformities due to degenerative conditions in those patients undergoing rTSA for the indication of an acute proximal humerus fracture. Over 92% of patients were noted to have a Walch type A glenoid, while 98% of patients had a Favard E0 morphology. This, in conjunction with osteoarthritis grading by the Samilson and Prieto classification, suggests that patients undergoing rTSA for fractures, at least in our cohort, were unlikely to have been impacted by severe degenerative joint disease prior to their injury.

With this lack of joint degeneration in mind, we also evaluated underlying glenoid morphology that could impact glenoid component placement. We found a median glenoid retroversion of 3 degrees. However, outcomes of rTSA may not be significantly impacted by version of the glenosphere component. As shown by Pettit et al. [30], there was no differences in improvement in ASES scores or range of motion comparing different preoperative Walch classifications, suggesting glenoid version may not have a significant impact on outcomes in rTSA. Placing a glenosphere in a more anteverted position, however, can result in improved internal rotation, though at the expense of external rotation [31].

**Fig. 5:**
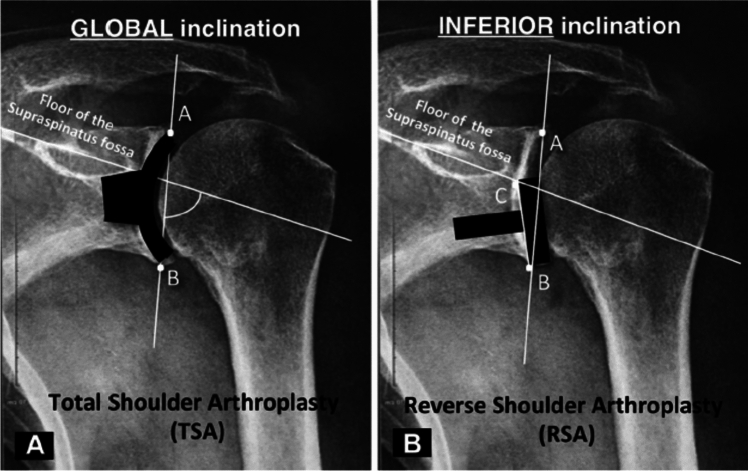
Placement of anatomic glenoid components typically utilize the entire glenoid face (**A**). However, in rTSA only the inferior portion of the glenoid is used, which can lead to inadvertent superior tilt of the baseplate unless accounted for when planning (**B**). Reprinted with permission from Boileau et al. [15]

While there does not seem to be consensus on the significance of glenosphere version on outcomes in rTSA, it is widely appreciated that glenosphere inclination is of great import. The RSA angle was developed to measure glenoid inclination as it applies to rTSA and help ensure optimal baseplate position, as traditional glenoid inclination, developed more for anatomic TSA, does not adequately consider the inferior portion of the glenoid and can lead to inadvertent superior tilt of the baseplate with certain glenoid morphologies **(**Fig. 5**)** [15]. Based on their findings, Boileau et al. [15] surmised that the coronal plane inclination of the inferior portion of the glenoid was on average 10° more than the entire glenoid fossa. In other words, the RSA angle is usually 10° more than the glenoid inclination, which can have tremendous implications for surgery. Thus, simply placing a guide that allows a pin to be placed in 10° of interior tilt, as is commonly available in many implant systems, may not be sufficient for ensuring that the glenosphere is placed without superior tilt. Dilisio et al. [32] has noted that this technique does not reliably result in correcting superior inclination and can also result in the reaming of significant inferior glenoid bone. Boileau et al. [15] also highlighted that it may be best to correct this superior inclination using bone graft or an augmented wedge-shaped baseplate.

Gutierrez et al. [33] recommended avoiding superior tilt as it could lead to scapular notching. Reviewing results with a minimum of 5 years follow-up, Deuthman et al. [34] found a 15% rate of severe scapular notching that was associated with glenoid superior inclination, although this did not appear to be correlated with the need for revision surgery. Randelli et al. [35] noted increased rates of joint instability in patients with superior tilt of the glenosphere when compared to those with 10 degrees of inferior tilt. In a study by Tashjian et al. [36], the authors found not only that instability was associated with increased superior tilt of the baseplate, but there was also an association with a greater relative increase in postoperative glenoid inclination compared to preoperative films.

In this study cohort, there was a median RSA angle of 19°, with over a third of patients measuring 20° or greater. This data compares favorably with other studies [15, 37]. Boileau et al. [15], who first described the RSA angle, reported a mean RSA angle of 20° ± 5°. It should be noted, however, that their readings were on patients with rotator cuff tear arthropathy, and, as a result, they had widespread variety of glenoid morphologies according to the Favard classification compared to this study.

**Fig. 6:**
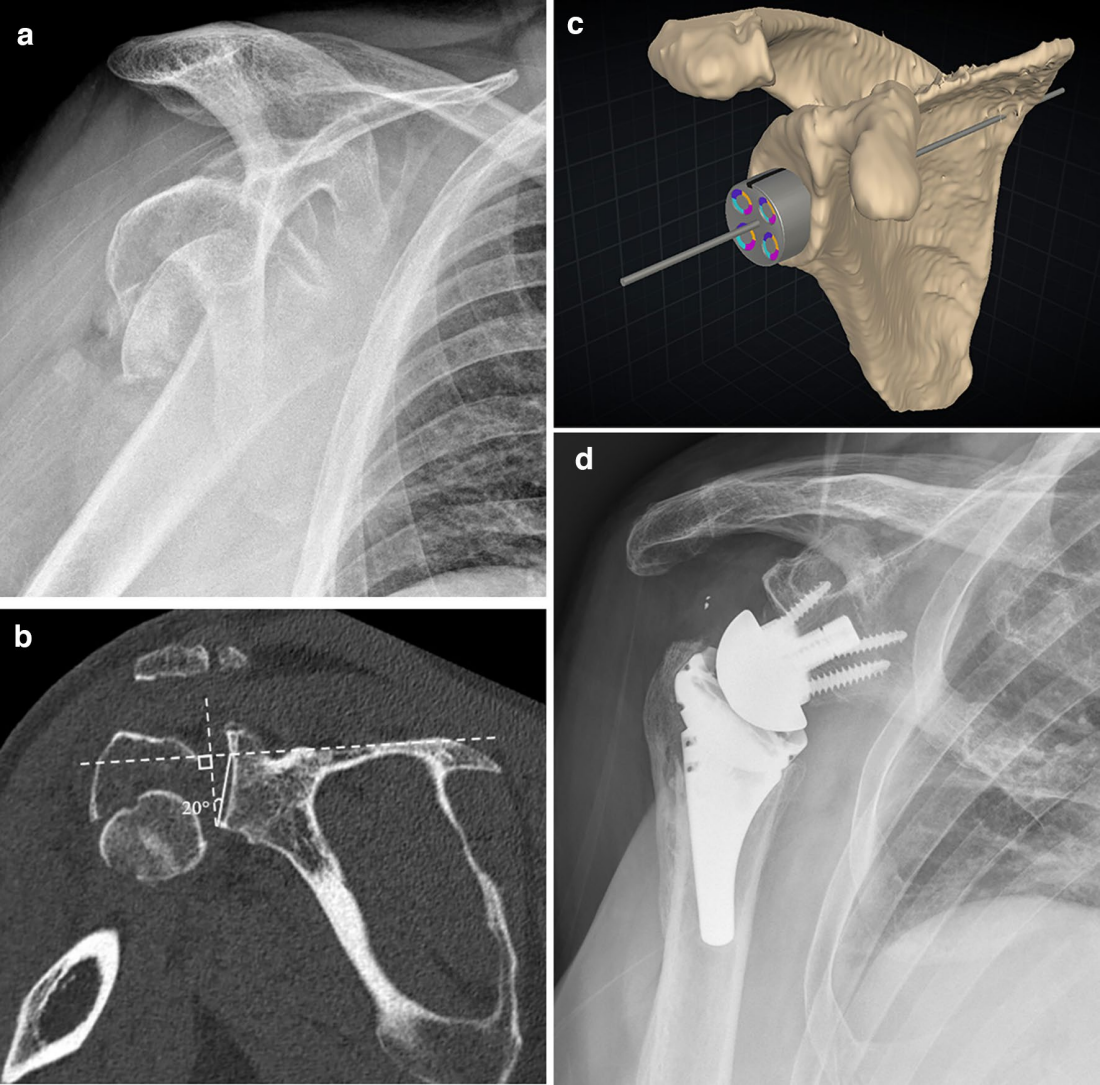
**A** Scapular Y view radiograph of the shoulder showing a comminuted proximal humeral surgical neck fracture with impaction, displacement, and extension through the greater tuberosity. **B** Coronal CT image through the scapula and glenohumeral joint in the same patient, again showing the comminuted, displaced proximal humeral fracture, as well as measurement of the RSA angle (20 degrees in this patient). **C** Reconstructed 3D image of the scapula from prior CT scan on the same patient with preoperative templating software used to determine glenoid baseplate position and size. Due to the patient’s anatomy and specifically the RSA angle, an augmented component was utilized. **D** Grashey radiograph of the right shoulder in the same patient status post rTSA placement demonstrates appropriate position and alignment of the prosthesis, specifically with neutral to slightly inferior alignment of the baseplate. Note is made of incidental metal debris in the subacromial area

With this knowledge, surgeons may want to consider using planning software to better evaluate glenoid anatomy, and, where deemed necessary, obtain a patient-specific guide when performing rTSA for a proximal humerus fracture. This can allow for better evaluation of glenoid anatomy and result in precise placement of the appropriate baseplate at surgery [18, 20, 21] **(**Fig. 6**)**. Iannoti et al. [18] previously demonstrated that glenoid inclination is often inadvertently under corrected by surgeons. They showed that surgeons will deviate from their preoperative plan on average by 10.7° in inclination, but that with 3-D planning software and a transfer device this deviation decreased to 2.8°.

**Fig. 7:**
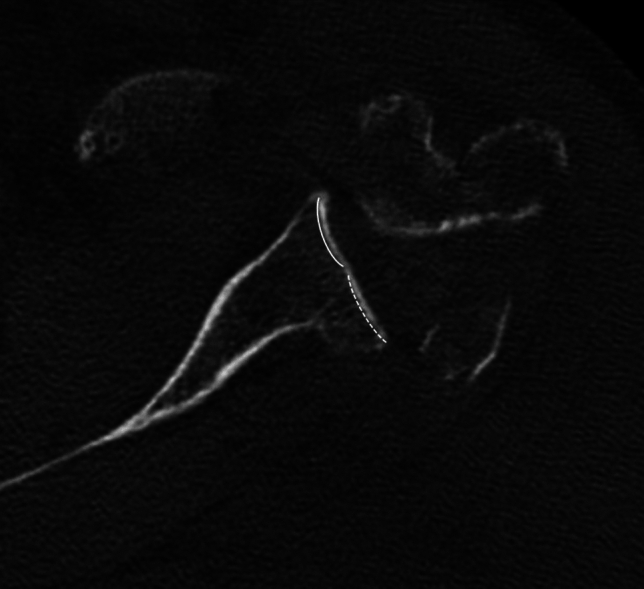
Axial CT image through the mid glenoid showing Walch class B2 morphology with a biconcave articular surface, including the expected concavity anteriorly (solid line) and secondary concavity posteriorly from osseous wear (dashed line)

One noted strength of the study is independent assessment of the images by two readers from different subspecialties who have expertise in shoulder musculoskeletal diseases. The equivalence test confirmed equivalent ratings and practicality measurement of preoperative glenoid version and RSA angle. This study does also have inherent limitations. It is a retrospective study, and with a cohort of just over fifty patients, there is the potential for the introduction of bias. Also, the study only included patients that underwent surgery, so it is difficult to note the morphological characteristics of all patients that sustain a proximal humerus fracture. Furthermore, classifications of glenoid morphology often take into account humeral head position in relation to the glenoid. In this study cohort, a significant number of patients sustained fracture dislocations, so glenoid morphology was determined without considering humeral head position (Fig. 7). However, the radiology literature does suggest that Walch classifications may be assigned by assessing the glenoid in isolation [38]. Lastly, patient reported outcomes of those patients without preoperative CT were not compared to those with preoperative studies.

## Conclusions

Currently, preoperative CT imaging is widely utilized for rTSA in the elective setting but may not be routinely performed in cases of trauma. Utilizing a preoperative CT in trauma patients to assess each patient’s glenoid anatomy, in addition to its benefit in assessing the anatomy of the fracture, may be warranted. While rates of glenoid deformity due to rotator cuff disease or osteoarthritis are low in patients presenting with a proximal humerus fracture, surgeons should consider taking into account glenoid version and, more importantly, RSA angle to allow for the optimal placement of the glenoid component. This can help treating surgeons to select appropriate components and better plan baseplate positioning, potentially reducing the risk of inadvertently placing the glenosphere with superior tilt.

## Data Availability

No datasets were generated or analysed during the current study.

## References

[1] Baron JA, Karagas M, Barrett J, Kniffin W, Malenka D, Mayor M et al (1996) Basic epidemiology of fractures of the upper and lower limb among Americans over 65 years of age. Epidemiology 7:612–618. 10.1097/00001648-199611000-00008 8899387 10.1097/00001648-199611000-00008

[2] Hanson B, Neidenbach P, de Boer P, Stengel D (2009) Functional outcomes after nonoperative management of fractures of the proximal humerus. J Shoulder Elbow Surg 18:612–621. 10.1016/j.jse.2009.03.024 19559373 10.1016/j.jse.2009.03.024

[3] Dillon MT, Prentice HA, Burfeind WE, Chan PH, Navarro RA (2019) The increasing role of reverse total shoulder arthroplasty in the treatment of proximal humerus fractures. Injury 50:676–680. 10.1016/j.injury.2019.01.034 30738568 10.1016/j.injury.2019.01.034

[4] Critchley O, McLean A, Page R, Taylor F, Graves S, Lorimer M et al (2020) Reverse total shoulder arthroplasty compared to stemmed hemiarthroplasty for proximal humeral fractures: a registry analysis of 5946 patients. J Shoulder Elbow Surg 29:2538–2547. 10.1016/j.jse.2020.04.005 32684280 10.1016/j.jse.2020.04.005

[5] Cuff DJ, Pupello DR (2013) Comparison of hemiarthroplasty and reverse shoulder arthroplasty for the treatment of proximal humeral fractures in elderly patients. J Bone Joint Surg 95:2050–2055. 10.2106/JBJS.L.01637 24257664 10.2106/JBJS.L.01637

[6] Gallinet D, Clappaz P, Garbuio P, Tropet Y, Obert L (2009) Three or four parts complex proximal humerus fractures: hemiarthroplasty versus reverse prosthesis: a comparative study of 40 cases. Orthop Traumatol Surg Res 95:48–55. 10.1016/j.otsr.2008.09.002 19251237 10.1016/j.otsr.2008.09.002

[7] Garrigues GE, Johnston PS, Pepe MD, Tucker BS, Ramsey ML, Austin LS (2012) Hemiarthroplasty versus reverse total shoulder arthroplasty for acute proximal humerus fractures in elderly patients. Orthopedics 35:e703-708. 10.3928/10477447-20120426-25 22588413 10.3928/01477447-20120426-25

[8] Sebastiá-Forcada E, Cebrián-Gómez R, Lizaur-Utrilla A, Gil-Guillén V (2014) Reverse shoulder arthroplasty versus hemiarthroplasty for acute proximal humeral fractures. A blinded, randomized, controlled, prospective study. J Shoulder Elbow Surg 23:1419–1426. 10.1016/j.jse.2014.06.035 25086490 10.1016/j.jse.2014.06.035

[9] Franceschi F, Giovannetti de Sanctis E, Gupta A, Athwal GS, Di Giacomo G (2023) Reverse shoulder arthroplasty: State-of-the-art. J ISAKOS 8:306–317. 10.1016/j.jisako.2023.05.007 37301479 10.1016/j.jisako.2023.05.007

[10] Frankle MA, Teramoto A, Luo ZP, Levy JC, Pupello D (2009) Glenoid morphology in reverse shoulder arthroplasty: classification and surgical implications. J Shoulder Elbow Surg 18:874–885. 10.1016/j.jse.2009.02.013 19482489 10.1016/j.jse.2009.02.013

[11] Levy JC, Virani N, Pupello D, Frankle M (2007) Use of the reverse shoulder prosthesis for the treatment of failed hemiarthroplasty in patients with glenohumeral arthritis and rotator cuff deficiency. J Bone Joint Surg Br 89:189–195. 10.1302/0301-620X.89B2.18161 17322433 10.1302/0301-620X.89B2.18161

[12] Werthel JD, Villard A, Kazum E, Deransart P, Ramierz O (2023) Accuracy of reverse shoulder arthroplasty angle according to the size of the baseplate. J Shoulder Elbow Surg 32:310–317. 10.1016/j.jse.2022.07.006 35998779 10.1016/j.jse.2022.07.006

[13] Young A, Walch G, Boileau P, Favard L, Gohlke F, Loew M et al (2011) A multicentre study of the long-term results of using a flat-back polyethylene glenoid component in shoulder replacement for primary osteoarthritis. J Bone Joint Surg Br 93:210–216. 10.1302/0301-620X.93B2.25086 21282761 10.1302/0301-620X.93B2.25086

[14] Zumstein MA, Pinedo M, Old J, Boileau P (2011) Problems, complications, reoperations, and revisions in reverse total shoulder arthroplasty: a systematic review. J Shoulder Elbow Surg 20:146–157. 10.1016/j.jse.2010.08.001 21134666 10.1016/j.jse.2010.08.001

[15] Boileau P, Gauci M, Wagner ER, Clowez G, Chaoui J, Chellli M et al (2019) The reverse shoulder arthroplasty angle: a new measurement of glenoid inclination for reverse shoulder arthroplasty. J Shoulder Elbow Surg 28:1281–1290. 10.1016/j.jse.2018.11.074 30935825 10.1016/j.jse.2018.11.074

[16] Walch G, Badet R, Boulahia A, Khoury A (1999) Morphologic study of the glenoid in primary glenohumeral osteoarthritis. J Arthroplasty 14:756–760. 10.1016/s0883-5403(99)90232-2 10512449 10.1016/s0883-5403(99)90232-2

[17] Favard L, Berhouet J, Walch G, Chaoui J, Lévigne C (2017) Superior glenoid inclination and glenoid bone loss: definition, assessment, biomechanical consequences, and surgical options. Orthopade 46:1015–1021. 10.1007/s00132-017-3496-1 29098355 10.1007/s00132-017-3496-1

[18] Iannotti J, Baker J, Rodriguez E, Brems J, Ricchetti E, Mesiha M et al (2014) Three-dimensional preoperative planning software and a novel information transfer technology improve glenoid component positioning. J Bone Joint Surg Am 96:e71. 10.2106/JBJS.L.01346 24806017 10.2106/JBJS.L.01346

[19] Tashjian RZ, Beck L, Stertz I, Chalmers PN (2021) Preoperative three-dimensional computer planning for reverse total shoulder arthroplasty and bone grafting for severe glenoid deformity. Shoulder Elbow 13:492–501. 10.1177/1758573220908903 34659482 10.1177/1758573220908903 PMC8512969

[20] Berhouet J, Gulotta LV, Dines DM, Craig E, Warren RF, Choi D et al (2017) Preoperative planning for accurate glenoid component positioning in reverse shoulder arthroplasty. Orthop Traumatol Surg Res 103:407–413. 10.1016/j.otsr.2016.12.019 28238965 10.1016/j.otsr.2016.12.019

[21] Werner BC, Denard PJ, Tokish JM, Bedi A, Donegan RP, Metcalfe N et al (2022) The addition of preoperative three-dimensional analysis alters implant choice in shoulder arthroplasty. Shoulder Elbow 14:378–384. 10.1177/1758573221989306 35846399 10.1177/1758573221989306 PMC9284305

[22] Bahrs C, Rolauffs B, Südkamp NP, Schmal H, Eingartner C, Dietz K et al (2009) Indications for computed tomography (CT-) diagnostics in proximal humerus fractures: a comparative study of plain radiography and computed tomography. BMC Musculoskeletal Disord 10:33. 10.1186/1471-2474-10-33 10.1186/1471-2474-10-33 PMC2678973 19341472

[23] Samilson RL, Prieto V (1983) Dislocation arthropathy of the shoulder. J Bone Joint Surg Am 65(4):456–460 6833319

[24] Coifman I, Brunner UH, Scheibel M (2022) Dislocation arthropathy of the shoulder. J Clin Med 11:2019. 10.3390/jcm11072019 35407627 10.3390/jcm11072019 PMC8999818

[25] Friedman RJ, Hawthorne KB, Genez BM (1992) The use of computerized tomography in the measurement of glenoid version. J Bone Joint Surg Am 74:1032–1037 1522089

[26] Bercik MJ, Kruse K 2nd, Yalizis M, Gauci MO, Chaoui J, Walch G (2016) A modification to the Walch classification of the glenoid in primary glenohumeral osteoarthritis using three-dimensional imaging. J Shoulder Elbow Surg 25:1601–1606. 10.1016/j.jse.2016.030.010 27282738 10.1016/j.jse.2016.03.010

[27] Favard L, Lautmann S, Sirveaux F, Oudet D, Kerjean Y, Huguet D (2001) Hemiarthroplasty versus reverse arthroplasty in the treatment of osteoarthritis with massive rotator cuff tear. In: Walch G, Boileau P, Molé D (eds) 2000 Shoulder Prostheses. Two to Ten Years Follow-Up. Sauramps Medical, Paris, pp 261–268.

[28] Sirveaux F, Favard L, Oudet D, Huquet D, Walch G, Molé D (2004) Grammont inverted total shoulder arthroplasty in the treatment of glenohumeral osteoarthritis with massive rupture of the cuff. Results of a multicentre study of 80 shoulders. J Bone Joint Surg Br 86:388–395. 10.1302/0301-620x.86b3.14024 15125127 10.1302/0301-620x.86b3.14024

[29] Rodriguez K, Levin J, Solomon J, Hurley ET, Lorenzana D, Samei E, et al (2024) Preoperative planning for shoulder arthroplasty is feasible with computed tomography at lower-than-conventional radiation doses. J Shoulder Elbow Surg Oct 21:S1058 2746(24)00769–9. Online ahead of print. 10.1016/j.jse.2024.08.038 10.1016/j.jse.2024.08.038 39442862

[30] Pettit RC, Saini SB, Puzzitiello RN, Hart PJ, Ross G, Kirsch JM et al (2022) Primary reverse total shoulder arthroplasty: does glenoid morphology matter? J Shoulder Elbow Surg 51:923–931. 10.1016/j.jse.2021.10.022 10.1016/j.jse.2021.10.022 34800669

[31] Huish EG Jr, Athwal GS, Neyton L, Walch G (2021) Adjusting implant size and position can improve internal rotation after reverse total shoulder arthroplasty in a three-dimensional computational model. Clin Orthop Relat Res 479:198–294. 10.1097/CORR.0000000000001526 33044311 10.1097/CORR.0000000000001526 PMC7899712

[32] Dilisio MF, Warner JJ, Walch G (2016) Accuracy of the subchondral smile and surface referencing techniques in reverse shoulder arthroplasty. Orthopedics 39:e615-620. 10.3928/01477-20160610-04 27322170 10.3928/01477447-20160610-04

[33] Gutiérrez S, Greiwe RM, Frankle MA, Siegal S, Lee WE 3rd (2007) Biomechanical comparison of component position and hardware failure in the reverse shoulder prosthesis. J Shoulder Elbow Surg 16:S9-12. 10.1016/j.jse.2005.11.008 16990024 10.1016/j.jse.2005.11.008

[34] Deuthman NC, Aibinder WR, Nguyen NTV, Sanchez-Sotelo J (2020) The influence of glenoid component position on scapular notching: a detailed radiographic analysis at midterm follow-up. JSES Int 4:144–150. 10.1016/j.jses.2019.11.004 10.1016/j.jses.2019.11.004 PMC7075770 32195477

[35] Randelli P, Randelli F, Arrigoni P, Ragone V, D’Ambrosi R, Masuzzo P, et al (2014) Optimal glenoid component inclination in reverse shoulder arthroplasty. How to improve implant stability. Musculoskelet Surg 98 Suppl 1:15–18. 10.1007/s12306-014-0324-1 10.1007/s12306-014-0324-1 24659201

[36] Tashjian RZ, Martin BI, Ricketts CA, Henninger HB, Granger EK, Chalmers PN (2018) Superior baseplate inclination is associated with instability after reverse total shoulder arthroplasty. Clin Orthop Relat Res 476:1622–1629. 10.1097/CORR.0000000000000340 29781910 10.1097/CORR.0000000000000340 PMC6259729

[37] Motta G, Amaral MV, Cohen M, Schiefer M, da Fonseca RS, Carolina Oliveira ACL (2024) Evaluation of the accuracy and interobserver agreement of the reverse shoulder angle in the preoperative planning of reverse total shoulder arthroplasties. Shoulder Elbow Nov 26:17585732241300686. Online ahead of print. 10.1177/17585732241300686 10.1177/17585732241300686 PMC11600422 39610691

[38] Sharifi A, Siebert MJ, Chhabra A (2020) How to measure glenoid bone stock and version and why it is important: A practical guide. Radiographics 40:1671–1683. 10.1148/rg.2020200008 33001780 10.1148/rg.2020200008

